# Effects of different metabolizable energy levels on apparent nutrient digestibility and metabolism, blood biochemical indicators, and fecal flora diversity in racing pigeons undergoing exercise training

**DOI:** 10.3389/fmicb.2025.1632529

**Published:** 2025-09-16

**Authors:** Xiao bin Li, Xin sheng Guo, Hai ying Li, Jia jia Liu, Jian wei Lin, Sheng chen Zheng, Li feng Ke

**Affiliations:** ^1^College of Animal Science, Xinjiang Agricultural University, Urumqi, China; ^2^Moyu Blue Sea Pigeon Industry Co., LTD., Hetian, Xinjiang, China; ^3^Yanqi County Lisheng Rare Poultry Breeding Professional Cooperative, Xinjiang, China

**Keywords:** pigeon, metabolizable energy, oxidative stress, serum biochemical variables, fecal microbiota

## Abstract

This study aimed to determine the optimal dietary energy requirements for pigeons undergoing exercise training. A total of 200 pigeons were randomly assigned to five groups (*n* = 40 per treatment) and subjected to 1 h of daily flight training. A one-way ANOVA design was employed, testing five dietary energy levels (12.03, 12.20, 12.32, 12.46, and 12.59 MJ/kg). The experiment lasted for 90 days. Results indicated that the metabolizable energy (ME) level significantly influenced nutrient digestion and metabolism, serum biochemical variables, and the microbial diversity and composition of exercise-trained pigeons. Specifically, the T5 group (12.59 MJ/kg) showed a significant increase in apparent organic matter (OM) digestion (*P* < 0.05), while the T4 group (12.46 MJ/kg) exhibited a significant increase in crude protein (CP) digestion (*P* < 0.01). Both the T2 and T5 groups demonstrated increased crude fat (EE) digestion (*P* < 0.05). Biochemical analysis revealed that the total protein (TP) and globulin (GLB) levels in the T1 group were significantly higher (*P* < 0.05 or *P* < 0.01). The T4 group showed elevated aspartate aminotransferase (AST) and alanine aminotransferase (ALB) levels (*P* < 0.05), while the T2 group exhibited significantly increased triglycerides (TG) and glucose (GLU) levels (*P* < 0.05 or *P* < 0.01). The T5 group had significantly higher catalase (CAT), superoxide dismutase (SOD), glutathione peroxidase (GSH-PX), and total antioxidant capacity (T-AOC; *P* < 0.05 or *P* < 0.01), whereas malondialdehyde (MDA) levels were significantly reduced (*P* < 0.01). Additionally, dietary ME levels affected microbial diversity and composition. The T1 group had higher abundance of Halobacterota and Verrucomicrobiota compared to the other groups (*P* < 0.05), while *Lactobacillus* abundance was greater in the T1 group than in the T3 group (*P* < 0.05). In contrast, *Clostridium*_*sensu*_*stricto_1* and *Romboutsia* were more abundant in the T3 group compared to the T1, T2, T4, and T5 groups (*P* < 0.05). The T5 group exhibited higher abundance of *Ligilactobacillus* than the T1 and T2 groups (*P* < 0.05). Correlation analysis revealed a significant positive relationship between MDA and Halobacterota, Halobacteria, Bacillales, Bacillaceae, Moraxellaceae, and Bacillus (*P* < 0.05). CAT was positively correlated with DNA metabolism, replication and repair, and nucleotide metabolism (*P* < 0.01), while T-AOC was positively associated with food synthesis, replication and repair, and glycan biosynthesis and metabolism (*P* < 0.05). GSH-PX was positively correlated with membrane transport, replication and repair, and nucleotide metabolism. MDA also showed a significant positive correlation with signal transduction (*P* < 0.05). In conclusion, the study indicates that ME levels ranging from 12.03 to 12.59 MJ/kg significantly influence nutrient digestion, metabolism, serum biochemistry, and microbial diversity in exercise-trained pigeons. For optimal nutrient requirements, health, gastrointestinal balance, and economic efficiency, a dietary ME level of 12.32–12.46 MJ/kg is recommended for practical pigeon production.

## 1 Introduction

The sport of pigeon racing originated and developed in Belgium in the 19th century (Marlier, 2022). Modern homing pigeon competitions consist of two main types: racing competitions and beauty contests. Racing competitions determine the winner based on the fastest homing time over different distances. Beauty contests are aesthetic competitions where winning racing pigeons are evaluated and ranked based on a comprehensive score of their body type, feather condition, head, etc. Racing competitions further include various formats such as homing races, challenge matches, and loft races.

In pigeon racing, prolonged flight training and competition impose significant load and stress on the pigeon's body, easily leading to cumulative fatigue, manifested as decreased physical strength, reduced athletic ability, and slower flight speeds. Flight training places stringent demands on the physiological functions of racing pigeons, among which the sustained and efficient supply and utilization of energy serve as the crucial material foundation determining training effectiveness and competition success. Bird flight muscles are the most energy consuming muscle movement in vertebrates, with the highest energy metabolism rate. Compared to moving mammals, flapping flight consumes more energy than running (Butler, 2016). During the flight of racing pigeons, their muscles mainly rely on lipid oxidation for energy supply, with fat accounting for over 60% of the energy supply and minimal changes in blood glucose concentration. Pigeons fly at maximum oxygen uptake ($\overset{∙}{V}O_{2}$, max) with a respiratory quotient (RQ) of 0.73, indicating dependence on fat oxidation (Stefaniuk-Szmukier et al., 2024). The flight power of birds is mainly provided by the pectoralis major muscle, which needs to contract continuously at high frequency during flight, resulting in a large workload and high energy cost. During flight, chest muscle contraction consumes a large amount of energy, and the rate of energy production determines the working ability of skeletal muscles. Energy supply is crucial for skeletal muscle contraction (Hargreaves and Spriet, 2020). Energy is a vital component of feed and the primary requirement for maintaining life. The survival, growth, development, and production of livestock and poultry all require energy participation, and the metabolic activities of various nutrients in feed are also regulated by energy. Dietary energy, existing primarily in the forms of fats, proteins, and carbohydrates, is a key factor in maintaining physiological activities such as growth, development, reproduction, lactation, and exercise in livestock and poultry (Noblet et al., 2001). Studies have shown (Xiang, 2024) that when dietary energy intake cannot meet the body's maintenance requirements, the body will catabolize its own energy reserves, resulting in energy loss; conversely, when dietary energy intake exceeds maintenance and production requirements, surplus energy is either stored as lipids in the body or excreted, leading to wastage of dietary energy (Birkett and Lange, 2001; Lizardo et al., 2002). It is evident that dietary energy levels that are either too high or too low are detrimental to the expression of growth performance, production performance, and athletic performance (Fagundes et al., 2009).

Therefore, delving into the supply strategies, nutrient metabolic pathways of energy for sport pigeons during the training adaptation period and under extreme competition conditions, and its intrinsic relationship with athletic performance (endurance, speed, homing rate), holds significant theoretical value not only for revealing the energy physiology mechanisms of this unique athletic model (the racing pigeon) but also provides critical scientific basis for optimizing their feeding management, training program formulation, and competition strategy adjustment. This can significantly enhance the competitive level of sport pigeons.

Different training intensities and different competition types lead to distinct energy requirements and metabolism in racing pigeons, but all increase the body's energy demands. It is hypothesized that dietary energy level affects the metabolism of dietary energy in racing pigeons, thereby influencing their athletic training level. What, then, is the appropriate metabolizable energy (ME) level? What impact does a sustained increase in ME level have on nutrient digestion and metabolism, blood physiology, intestinal microbiota, etc., in training pigeons? Consequently, this study uses pigeons subjected to daily regulated flight training of 1 h as the research subjects. By feeding diets with different energy levels, it investigates their effects on nutrient digestibility and metabolism, blood biochemical parameters, antioxidant capacity, blood gas parameters, and fecal microbial diversity in training pigeons. The aim is to provide a reference basis for the precise dietary formulation of pigeons undergoing athletic training.

## 2 Materials and methods

### 2.1 Ethical statement

All experimental procedures, management, and bird husbandry followed the regulations and instructions of the Animal Welfare and Ethics Committee of Xinjiang Agricultural University, Urumqi, Xinjiang, China (Approval number 2019004).

### 2.2 Pigeon care and experimental design

This study depended on mixed-sex (1:1), 12-month-old pigeons. Pigeons were obtained from the poultry facility, Yanqi County Li Feng rare poultry breeding professional cooperative, Xinjiang, China. To control the dietary adaptation of pigeons, they have a 2-week adaptation period. During the first week of the adaptation period, they transition from the original diet to the experimental diet in a 1:1 ratio and gradually transition to the 100% experimental diet in the second week. The trial lasted for 90 days. Each pigeon was individually weighed with a mean body weight of 340.69 ± 39.30 g. A total of 200 pigeons were randomly divided into five treatment groups (10 replicates per group, four birds per replicate). The groups were fed basal diets with graded metabolizable energy (ME) levels: T1 received 12.03 MJ/kg ME, T2 received 12.20 MJ/kg ME, T3 received 12.32 MJ/kg ME, T4 received 12.46 MJ/kg ME, and T5 received 12.59 MJ/kg ME. Throughout the experiment, all pigeons were housed under identical conditions with free access to water and provided 20 g/day of the basal diet (composition and nutritional levels detailed in Table 1). The loft temperature was maintained at 24–30 °C under natural lighting without artificial supplementation. Adequate perches (≥20 cm per bird) were provided in a loft measuring 5 × 4 × 3 m, and the trial duration was 90 days. The experimental pigeons underwent daily flight training at 17:00, conducted in groups within a circular open-field testing area with a diameter of 200 m for free flight. The experimenters simultaneously applied two external signals: a visual signal (flag-waving) and an auditory signal (whistle sound). The stimulation protocol was continuously applied from the start of release until the test group had flown for 1 h. The flight duration was defined as the interval from the moment of take-off to the moment of landing.

**Table 1:** Nutrient levels of experimental diets for pigeons.

**Composition**	**T1**	**T2**	**T3**	**T4**	**T5**
Corn	66	65.5	65	64.5	64
Soybeanmeal	20	20	20	20	20
Secondary powder	5	5	5	5	5
Cotton protein	5	5	5	5	5
Soybean oil	0	0.5	1	1.5	2
Limestone (0–2 mm)	1	1	1	1	1
Limestone (2–4 mm)	1	1	1	1	1
2% Premix	2	2	2	2	2
Total	100	100	100	100	100
**Nutrient levels**					
DM (%)	91.40	92.19	91.96	92.07	91.75
OM (%)	96.05	96.25	96.18	96.30	96.42
GE/MJ	0.290	0.294	0.298	0.302	0.306
ME MJ/kg	12.03	12.20	12.32	12.46	12.59
CP (%)	18.43	18.38	18.34	18.47	18.23
EE (%)	2.17	1.99	2.00	2.23	2.19
CF (%)	3.07	3.06	3.05	3.04	3.03
Ca (%)	0.94	0.94	0.94	0.94	0.94
P (%)	0.58	0.58	0.58	0.58	0.58
Lys (%)	0.86	0.86	0.86	0.86	0.86
Met (%)	0.44	0.44	0.44	0.44	0.44

The premix provides the following per kilogram of the diet: Vitamin A 5,330 IU; Vitamin D3 1,300 IU; Vitamin E 30 mg; Vitamin K3 3.10 mg; Vitamin B1 2.00 mg; Vitamin B2 7.00 mg; Biotin 0.10 mg; Folic acid 1.30 mg; Niacin 37 mg; Calcium pantothenate 2,300 mg; Lysine 5,400 mg; Methionine 2,300 mg.

DM, OM, GE, ME, CP, EE, and CF are measured values, while the rest are calculated values.

### 2.3 Sample collection and index determination

#### 2.3.1 Nutrient digestion and metabolism sample collection and index determination

During the digestion and metabolism period, the feces of the test pigeons were collected by the full collection method. During the final week of the trial, each treatment group was housed in metabolic cages on a per-replicate basis, with four pigeons per cage. Fecal samples from the four pigeons within each replicate were pooled into one composite sample for subsequent analysis, resulting in 10 samples per group. After a 12 h fasting period, all pigeons were fed the experimental diet at 20 g/day for three consecutive days. Fecal samples from each cage were collected daily throughout this period and pooled by cage. Both diet samples and air-dried excreta samples were oven-dried at 65 °C, ground, and analyzed as follows.

Air-dried excreta samples were analyzed for dry matter (DM), organic matter (OM), metabolizable energy (ME), crude protein (CP), and ether extract (EE) content. Diet samples were analyzed for dry matter (DM), organic matter (OM), metabolizable energy (ME), crude protein (CP), ether extract (EE) content, and crude fiber (CF) content. Dry matter (DM) refers to GB/T 6435-2014, organic matter (OM) refers to GB/T 6438-2007, total energy (GE) refers to GB/T 14489.1-2008, crude protein (CP) refers to GB/T 6432-2018, ether extract (EE) refers to GB/T 6433-2006, the formula for calculating metabolic energy is ME (kcal/kg DM) = [GE intake – (GE feces + GE urine)]/Feed DM intake.

#### 2.3.2 Serum biochemical variables

On the final day of the experiment, blood samples were collected using non-heparinized syringes and placed in sterile vials. The samples were centrifuged at 3,000 rpm for 10 min to separate serum, which was then stored at −20 °C for further biochemical analysis. Serum samples from four pigeons within each replicate were pooled into one composite sample, resulting in 10 samples per group.

The measured serum biochemical indices included glutathione peroxidase (GSH-Px), superoxide dismutase (SOD), catalase (CAT), total antioxidant capacity (T-AOC), malondialdehyde (MDA), total protein (TP), albumin (ALB), alanine aminotransferase (ALT), aspartate aminotransferase (AST), urea, creatinine, and lipid profiles such as total cholesterol (TC), triglycerides (TG), low-density lipoprotein (LDL), and high-density lipoprotein (HDL). Serum globulin (GLB) levels were calculated as the difference between TP (biuret method) and ALB (bromocresol green method) using readings from the same sample.

All analyses were performed with a UV-Vis spectrophotometer (Agilent Technologies, model: Cary 5000) and a fully automated biochemical analyzer (Mindray Bio-Medical Electronics Co., Ltd., model: BS-240). Commercial assay kits purchased from Nanjing Jiancheng Bioengineering Institute were used according to the manufacturer's protocols.

#### 2.3.3 Blood gas analysis

Blood gas refers to a group of clinical indicators that evaluate the body 's respiratory function, acid-base balance, and electrolyte levels by detecting the concentrations of dissolved oxygen (O_2_), carbon dioxide (CO_2_), and related metabolites in arterial or venous blood. Before feeding on the last day of the experiment, blood samples were obtained via venipuncture of the brachial wing vein using 1 ml non-heparinized syringes and needles. Each four pigeon blood samples were combined into 1 sample for the determination, with 10 samples in each group. The measurement of blood gas indicators was conducted using a veterinary blood gas immunoanalyzer (Chengdu Seamaty Technology Co., Ltd., model: VG2, test cartridge: dry electrochemical method). Following the manufacturer's recommendations, the collected blood sample was loaded into the test cartridge. The test cartridge was immediately inserted into the device. Fourteen blood gas and chemical parameters were collected through the blood gas analyzer: pH, partial pressureof carbon dioxide (pCO_2_ mm Hg), partial pressure of oxygen (pO_2_, mm Hg), sodium (Na, mmol/L), potassium (K, mmol/L), Cl (mmol/L), ionized calcium (iCa), packed cell volume (PCV%), total concentration of carbon dioxide (TCO_2_, mmol/L), bicarbonate (HCO^3−^, mmol/L), hemoglobin (Hb, g/dl), extracellular fluid base excess[BE (ecf) mmol/L], base excess [BE (b) mmol/L], oxygen saturation (sO_2_%). Average total time for each sampling per pigeon was between 2 and 3 min. Test results were subsequently uploaded to a computer to allow statistical analysis of the data.

### 2.4 Microbial diversity and composition

On the final day of the trial, 50 fecal samples (collected from 40 pigeons per group, with four pigeons per replicate pooled into one sample) were analyzed for microbial diversity and composition via 16S rRNA sequencing. Total genomic DNA was extracted using the QIAamp Fast DNA Stool Mini Kit (Qiagen, Tianjin, China). The V3–V4 hypervariable regions of the bacterial 16S rRNA gene were amplified with specific primers 338F (5′-ACTCCTACGGGAGGCAGCAG-3′) and 806R (5′-GGACTACHVGGGTWTCTAAT-3′). Libraries were constructed using the NEBNext^®^ UltraTM IIDNA Library Prep Kit. Constructed libraries underwent Qubit and PCR quantification. After passing quality control, sequencing was performed on the No-vaSeq 6000 platform.

Raw sequencing reads were processed through the following steps: quality control was performed using fastp (v0.20.0; https://github.com/OpenGene/fastp), paired-end read assembly was conducted with FLASH (v1.2.7; http://www.cbcb.umd.edu/software/flash), and denoising was implemented via the DADA2 plugin in the QIIME2 pipeline (default parameters) to generate amplicon sequence variants (ASVs). Taxonomic classification of ASVs was performed using the Naive Bayes classifier in QIIME2 against the SILVA 16S rRNA gene database (v138).

Alpha diversity was assessed using the Chao1, Shannon, Simpson, Dominance, Goods_coverage, Observed_features, and Pielou_e indices, and calculated through Mothur (http://www.mothur.org/wiki/Calculators). Beta diversity was assessed through principal coordinate analysis (PCoA) based on Bray–Curti's dissimilarity. Differential taxa between groups were identified by linear discriminant analysis (LDA) with an effect size threshold of 3.5, followed by linear discriminant analysis effect size (LEfSe) analysis.

### 2.5 Statistical analysis

After preprocessing with Excel 2003, the apparent digestion and metabolism of nutrients, serum biochemical indicators, serum antioxidant capacity, and blood gas in sports pigeons were analyzed using SPSS 26.0 for one-way analysis of variance. Duncan's method was used for multiple comparisons between groups. All results are presented as mean ± standard deviation. *P* < 0.05 is considered significant, and *P* < 0.01 is considered extremely significant. Linear and quadratic regression methods were employed to analyze the corresponding data.

## 3 Results

### 3.1 Effects of different metabolizable energy levels on the apparent digestion and metabolism of nutrients in exercise-trained pigeons

The effects of different ME levels on the apparent digestion and metabolism of nutrients in exercise-trained pigeons are summarized in Table 2. From the table, it is evident that the ME level of the diet in group T5, which was 12.59 MJ/kg, significantly enhanced the organic matter digestibility in exercise-trained pigeons compared to those fed with a diet containing 12.46 MJ/kg ME (*P* < 0.05). The crude protein digestibility in group T3 was markedly higher than in the other groups (T1, T4, and T5) with extreme significance (*P* < 0.01). The fat digestibility in groups T2 and T5 was significantly greater than in group T4 (*P* < 0.05). There was no significant difference in GE and ME of exercise training pigeons in diets with different ME levels (*P* < 0.05).

**Table 2:** Effects of different metabolizable energy levels on the apparent digestion and metabolism of nutrients in exercise-trained pigeons.

**Item**	**T1**	**T2**	**T3**	**T4**	**T5**
DM %	71.57 ± 5.62	72.32 ± 3.86	72.12 ± 3.67	69.81 ± 1.91	71.82 ± 3.57
OM %	76.61 ± 4.64^ab^	75.55 ± 3.85^ab^	77.46 ± 2.73^ab^	74.54 ± 2.07^b^	78.36 ± 2.41^a^
CP %	51.7 ± 9.93^Bb^	54.01 ± 9.12^ABb^	61.68 ± 3.58^Aa^	52.35 ± 4.71^Bb^	50.9 ± 3.85^Bb^
ME MJ/kg	12.03 ± 0.62	12.20 ± 0.81	12.32 ± 0.44	12.46 ± 0.37	12.59 ± 0.55
GE MJ/kg	75.21 ± 3.88	76.49 ± 5.09	77.04 ± 2.73	75.76 ± 2.27	76.32 ± 3.34
EE %	84.41 ± 10.20^ab^	87.08 ± 9.52^a^	79.69 ± 12.06^ab^	75.97 ± 14.31^b^	87.17 ± 6.86^a^

Data in the same column without letters or the same letter superscripts mean no significant difference (P > 0.05), different letter superscripts mean significant difference (P < 0.01 as highly significant, P < 0.05 as significant). Values with different lowercase superscript letters indicate a significant difference (P < 0.05), and values with different uppercase superscript letters indicate an extremely significant difference (P < 0.01).

### 3.2 Effects of different metabolizable energy levels in the diet on the serum biochemical indexes of exercise-trained pigeons

**Figure 1:**
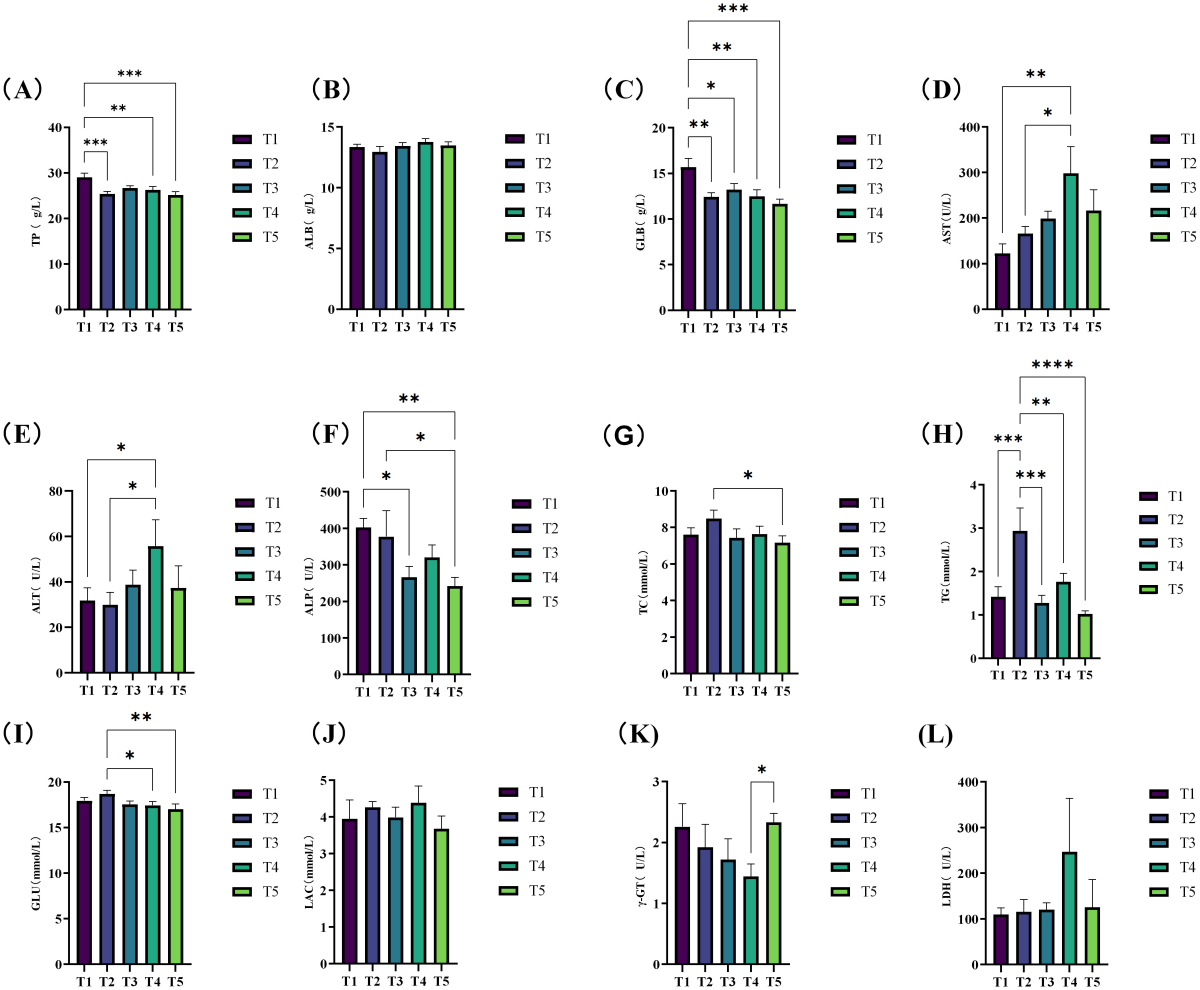
Effects of varying dietary metabolizable energy levels on serum biochemical indices of exercise-trained pigeons **(A–L)**. **(A)** TP, **(B)** ALB, **(C)** GLB, **(D)** AST, **(E)** ALT, **(F)** ALP, **(G)** TC, **(H)** TG, **(I)** GLU, **(J)** LAC, **(K)** γ-GT, **(L)** LDH. Asterisk “*” denotes a significant difference between groups (LDS test, *P* < 0.05), while “**” and higher indicate an extremely significant difference between groups (LDS test, *P* < 0.01). T1: 12.03 MJ/kg dietary metabolizable energy; T2: 12.20 MJ/kg dietary metabolizable energy; T3: 12.32 MJ/kg dietary metabolizable energy; T4: 12.46 MJ/kg dietary metabolizable energy; T5: 12.59 MJ/kg dietary metabolizable energy.

The effects of varying ME levels on the serum biochemical indices of exercise-trained pigeons are shown in Figure 1. The data indicate that different ME levels impact the serum biochemical parameters of pigeons to varying extents. Specifically, when the ME level was 12.03 MJ/kg, the serum total protein (TP) (Figure 1A) and globulin (Figure 1C) levels significantly increased compared to other groups (*P* < 0.05). Additionally, the levels of serum aspartate aminotransferase (AST; Figure 1D) and alanine aminotransferase (ALT; Figure 1E) were significantly higher in group T4 (12.46 MJ/kg) than in groups T1 and T2 (*P* < 0.05), with these enzyme levels increasing progressively with higher ME in the diet. Moreover, the serum alkaline phosphatase (ALP) levels (Figure 1F) in pigeons from groups T1 (12.03 MJ/kg) and T2 (12.20 MJ/kg) were significantly higher compared to other groups (*P* < 0.05). Notably, the plasma triglyceride (TG) levels (Figure 1H) in group T2 were extremely significantly higher than those in groups T1, T3, T4, and T5 (*P* < 0.01). Finally, the glucose (GLU) content (Figure 1I) was significantly higher in group T2 compared to group T4 (*P* < 0.05) and extremely significantly higher compared to group T5 (*P* < 0.01).

### 3.3 Effects of different metabolizable energy levels in the diet on the serum antioxidant capacity of exercise-trained pigeons

**Figure 2:**
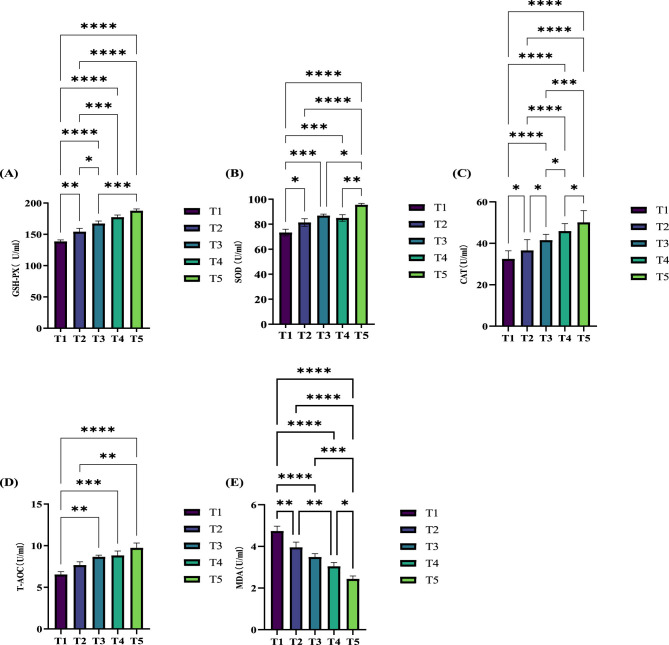
Effects of varying dietary metabolizable energy levels on the serum antioxidant capacity of exercise-trained pigeons **(A–E)**. **(A)** GSH-PX, **(B)** SOD, **(C)** CAT, **(D)** T-AOC, **(E)** MDA. Asterisk “*” denotes a significant difference between groups (LDS test, *P* < 0.05), while “**” and higher indicate an extremely significant difference between groups (LDS test, *P* < 0.01). T1: 12.03 MJ/kg dietary metabolizable energy; T2: 12.20 MJ/kg dietary metabolizable energy; T3: 12.32 MJ/kg dietary metabolizable energy; T4: 12.46 MJ/kg dietary metabolizable energy; T5: 12.59 MJ/kg dietary metabolizable energy.

The effects of different ME levels on the serum antioxidant capacity of exercise-trained pigeons are illustrated in Figure 2. The data show that varying ME levels in the diet significantly influence the serum antioxidant capacity of the pigeons. Specifically, the enzyme activity levels of serum glutathione peroxidase (GSH-PX) (Figure 2A), catalase (CAT; Figure 2C), and total antioxidant capacity (T-AOC) (Figure 2D) all increased significantly (*P* < 0.05) or extremely significantly (*P* < 0.01) as the ME level in the diet increased. At the 12.20 MJ/kg ME level, serum superoxide dismutase (SOD) activity (Figure 2B) increased significantly (*P* < 0.05). When the ME levels were 12.32, 12.46, and 12.59 MJ/kg, SOD activity increased extremely significantly (*P* < 0.01). Furthermore, compared to group T1, serum malondialdehyde (MDA) levels (Figure 2E) decreased significantly or extremely significantly (*P* < 0.05, *P* < 0.01) with increasing ME levels.

### 3.4 Effects of different metabolizable energy levels in the diet on blood gas of exercise-trained pigeons

The effects of varying ME levels in the diet on the blood gas parameters of exercise-trained pigeons are summarized in Table 3. As demonstrated, different ME levels significantly influence the blood gas profile of these pigeons. Specifically, at ME levels of 12.32 and 12.46 MJ/kg, pH, extracellular alkali reserve, and residual alkali levels in the blood showed a marked increase (*P* < 0.01) compared to other energy levels (12.03, 12.20, and 12.59 MJ/kg). At a diet ME level of 12.59 MJ/kg, the oxygen partial pressure in exercise-trained pigeons was significantly higher than that observed at 12.03 MJ/kg (*P* < 0.01). Blood Na^+^ concentrations in pigeons from groups T2 and T3 were significantly elevated compared to those in group T1 (*P* < 0.05). Furthermore, blood K^+^ levels in pigeons from group T5 were significantly higher than in groups T3 and T4 (*P* < 0.05). At energy levels of 12.03, 12.20, and 12.59 MJ/kg, blood Ca^2+^ concentrations showed a substantial increase (*P* < 0.01). Additionally, hemoglobin levels in pigeons from group T2 were significantly higher when the diet's ME level was 12.59 MJ/kg, compared to those in group T2 (*P* < 0.01). Likewise, oxygen saturation in pigeons from group T1 was extremely significantly increased at 12.59 MJ/kg compared to group T1 (*P* < 0.01).

**Table 3:** Effects of different dietary metabolic energy levels on blood gas of exercise–trained pigeons.

**Item**	**T1**	**T2**	**T3**	**T4**	**T5**
pH	7.38 ± 0.05^Bb^	7.41 ± 0.05^Bb^	7.48 ± 0.06^Aa^	7.50 ± 0.04^Aa^	7.41 ± 0.03^Bb^
Carbon dioxide partial pressure	39.36 ± 4.72^Aa^	34.42 ± 4.86^Bb^	35.05 ± 1.50^ABb^	35.15 ± 3.48^ABb^	33.29 ± 3.17^Bb^
Oxygen partial pressure	35.30 ± 6.60^Bb^	37.9 ± 3.76^ABb^	39.80 ± 3.82^ABb^	38.70 ± 4.30^ABb^	52.30 ± 24.22^Aa^
Na^+^	148.12 ± 1.99^b^	150.42 ± 1.47^a^	150.67 ± 2.12^a^	148.67 ± 2.08^ab^	150.16 ± 3.037^ab^
K^+^	2.34 ± 0.48^ab^	2.44 ± 1.36^ab^	1.90 ± 0.30^b^	2.02 ± 0.51^b^	2.95 ± 0.99^a^
Cl^−^	118.94 ± 4.23	118.86 ± 8.37	114.09 ± 2.46	114.69 ± 2.63	115.76 ± 5.10
Ca^2+^	1.26 ± 0.06^Aa^	1.29 ± 0.06^Aa^	1.19 ± 0.05^Bb^	1.15 ± 0.02^Bb^	1.26 ± 0.06^Aa^
Hematocrit	53.40 ± 2.37^ab^	48.40 ± 7.72^ab^	51.00 ± 5.59^ab^	47.00 ± 16.35^b^	55.60 ± 1.35^a^
Total carbon dioxide	24.33 ± 2.21	22.75 ± 3.25	27.15 ± 2.78	49.68 ± 65.57	21.95 ± 2.09
Hemoglobin	17.99 ± 0.57^ABab^	16.43 ± 2.64^Bb^	17.40 ± 1.91^ABab^	17.71 ± 1.35^ABab^	18.89 ± 0.48^Aa^
Bicarbonate	23.15 ± 2.12^BCb^	21.74 ± 3.13^Cb^	26.1 ± 2.81^ABa^	27.36 ± 3.97^Aa^	20.95 ± 2.06^Cb^
Extracellular liquid alkali residue	−1.74 ± 2.52^Bb^	−2.92 ± 3.42^Bb^	2.58 ± 3.73^Aa^	4.14 ± 4.51^Aa^	−3.38 ± 2.52^Bb^
Residual alkali in blood	−0.37 ± 1.16^Bb^	0.16 ± 0.95^Bb^	2.09 ± 1.60^Aa^	2.69 ± 1.42^Aa^	0.17 ± 0.74^Bb^
Oxygen saturation	65.60 ± 13.67^Bc^	72.558 ± 7.08^ABbc^	78.32 ± 6.11^Aab^	77.93 ± 5.60^Aab^	82.91 ± 6.93^Aa^

^A, B, C^Different superscripts indicate significant differences within a row (P < 0.01). ^a, b, c^Different superscripts indicate significant differences within a row (P < 0.05). T1: dietary metabolizable energy level is 12.03 MJ/kg; T2: dietary metabolizable energy level is 12.20 MJ/kg; T3: dietary metabolizable energy level is 12.32 MJ/kg; T4: dietary metabolizable energy level is 12.46 MJ/kg; T5: dietary metabolizable energy level is 12.59 MJ/kg.

### 3.5 Effects of different metabolizable energy levels in the diet on the α-diversity of fecal flora in exercise-trained pigeons

The effects of different ME levels in the diet on the α-diversity of fecal microbiota in exercise-trained pigeons are presented in Table 4. As shown, varying ME levels significantly influence the diversity of fecal microbiota in these pigeons. Notably, at an ME level of 12.03 MJ/kg, both the Chao1 and observed_features indices of fecal microbiota were significantly higher compared to groups T3 and T5 (*P* < 0.05), and extremely significantly higher than those in group T4 (12.46 MJ/kg) (*P* < 0.01). The Pielou_e index in group T4 was extremely significantly higher than in groups T1 and T2 (*P* < 0.01).

**Table 4:** Effects of different metabolizable energy levels in the diet on the alpha diversity of fecal flora in athletic training pigeons.

**Item**	**T1**	**T2**	**T3**	**T4**	**T5**
chao1	143.20 ± 93.79^Aa^	116.58 ± 83.96^ABab^	83.01 ± 22.51^ABb^	58.41 ± 10.83^Bc^	77.43 ± 33.17^ABb^
Dominance	0.20 ± 0.15	0.22 ± 0.10	0.17 ± 0.07	0.14 ± 0.05	0.17 ± 0.07
Goods_coverage	1.00 ± 0.00	1.00 ± 0.00	1.00 ± 0.00	1.00 ± 0.00	1.00 ± 0.00
Observed_features	141.4 ± 92.59^Aa^	114.8 ± 82.82^ABab^	82.2 ± 22.01^ABbc^	56.9 ± 9.11^Bc^	76.7 ± 32.52^ABbc^
Pielou_e	0.50 ± 0.11^Bb^	0.48 ± 0.10^Bb^	0.54 ± 0.05^ABab^	0.61 ± 0.07^Aa^	0.56 ± 0.07^ABab^
Shannon	3.45 ± 0.99	3.14 ± 0.78	3.44 ± 0.45	3.54 ± 0.36	3.47 ± 0.42
Simpson	0.80 ± 0.15	0.78 ± 0.10	0.83 ± 0.07	0.86 ± 0.05	0.83 ± 0.07

^A, B, C^ Different superscripts indicate significant differences within a row (P < 0.01). ^a, b, c^ Different superscripts indicate significant differences within a row (P < 0.05). T1: dietary metabolizable energy level is 12.03 MJ/kg; T2: dietary metabolizable energy level is 12.20 MJ/kg; T3: dietary metabolizable energy level is 12.32 MJ/kg; T4: dietary metabolizable energy level is 12.46 MJ/kg; T5: dietary metabolizable energy level is 12.59 MJ/kg.

### 3.6 Effects of different metabolizable energy levels in the diet on the β-diversity of fecal flora in exercise-trained pigeons

**Figure 3:**
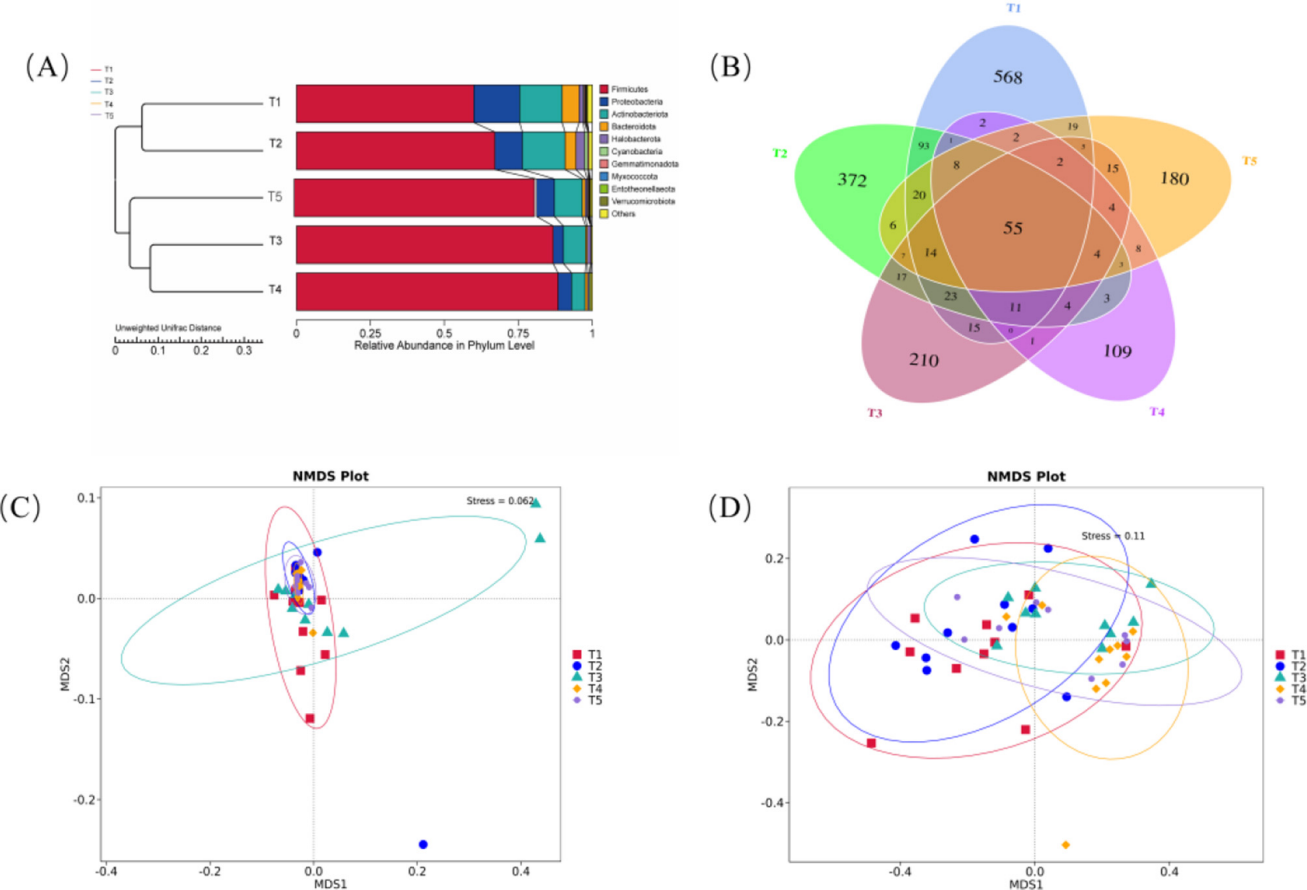
Effects of varying dietary metabolizable energy levels on the β-diversity of fecal flora in exercise-trained pigeons **(A–D)**. **(A)** UPGMA clustering tree at the phylum level, **(B)** Venn diagram, **(C, D)** Principal Coordinate analysis (PCoA). T1: 12.03 MJ/kg dietary metabolizable energy; T2: 12.20 MJ/kg dietary metabolizable energy; T3: 12.32 MJ/kg dietary metabolizable energy; T4: 12.46 MJ/kg dietary metabolizable energy; T5: 12.59 MJ/kg dietary metabolizable energy.

The effects of different ME levels on the β-diversity of fecal microbiota in exercise-trained pigeons are illustrated in Figure 3. The Venn diagram and UPGMA clustering tree analyses are presented in Figure 3B, where the Venn diagram shows 55 common OTUs across all five treatment groups. The number of unique OTUs was 568 in group T1, 372 in group T2, 210 in group T3, 109 in group T4, and 188 in group T5, indicating that group T1 harbored the highest diversity of bacterial species. Principal component analysis (PCA) plots further reveal that the goodness-of-fit in Figure 3C (Stress = 0.0062) is substantially better than that in Figure 3D (Stress = 0.11). These stress values, obtained through non-metric multidimensional scaling (NMDS), reflect the model's fit to the dataset—lower stress values indicate better model alignment with the data. Under the conditions in Figure 3C, the similarities and differences among the microbiota were more distinctly separated, with these differences closely associated with variations in the ME levels.

### 3.7 Effects of different metabolizable energy levels in the diet on the relative abundance of fecal flora in exercise-trained pigeons

**Figure 4:**
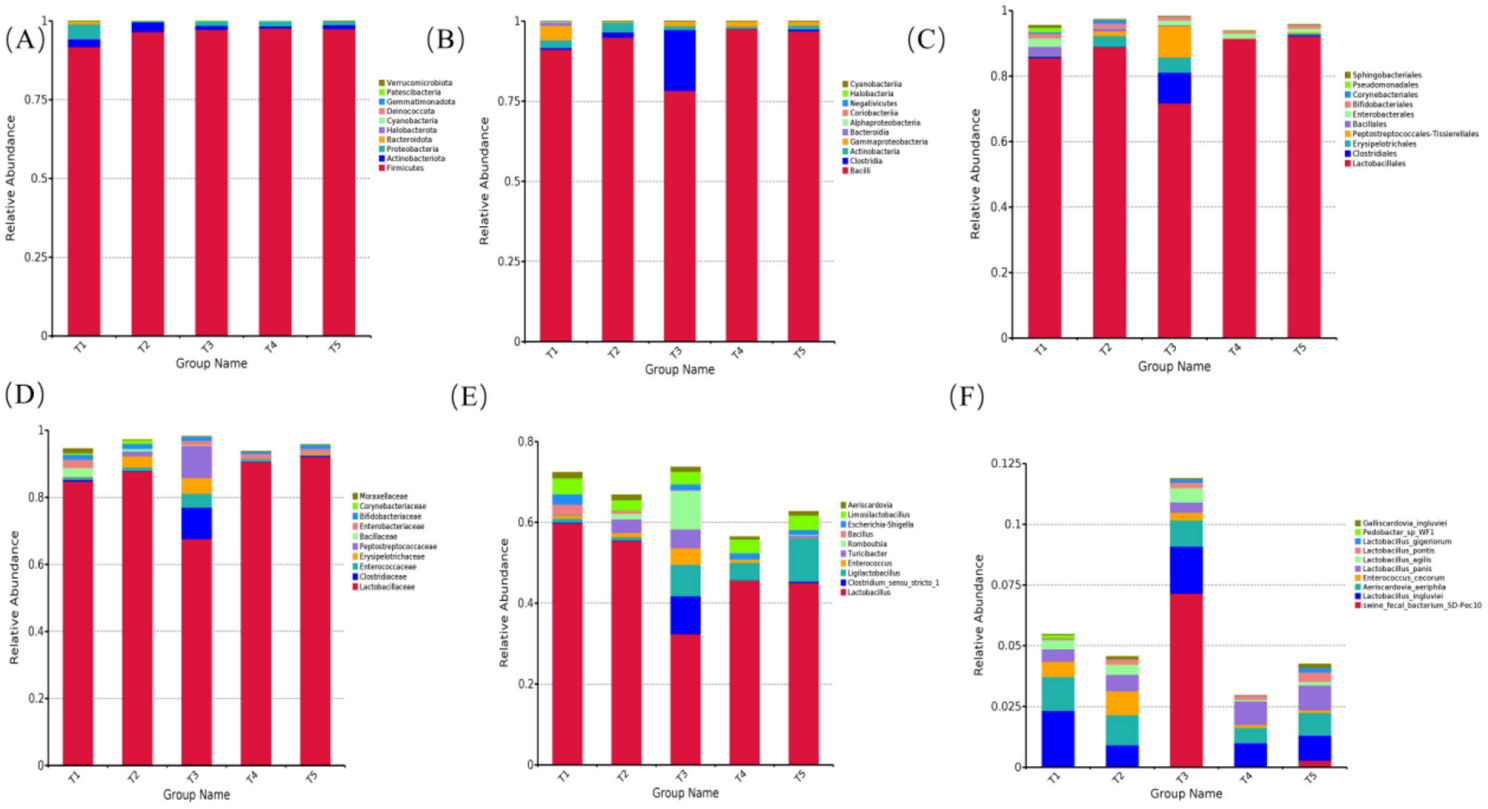
Effects of varying dietary metabolizable energy levels on the relative abundance of fecal flora in exercise-trained pigeons. **(A)** Column chart depicting species abundance at the phylum level. **(B)** Column chart depicting species abundance at the class level. **(C)** Column chart depicting species abundance at the order level. **(D)** Column chart depicting species abundance at the family level. **(E)** Column chart depicting species abundance at the genus level. **(F)** Column chart depicting species abundance at the species level. T1: 12.03 MJ/kg dietary metabolizable energy; T2: 12.20 MJ/kg dietary metabolizable energy; T3: 12.32 MJ/kg dietary metabolizable energy; T4: 12.46 MJ/kg dietary metabolizable energy; T5: 12.59 MJ/kg dietary metabolizable energy.

**Figure 5:**
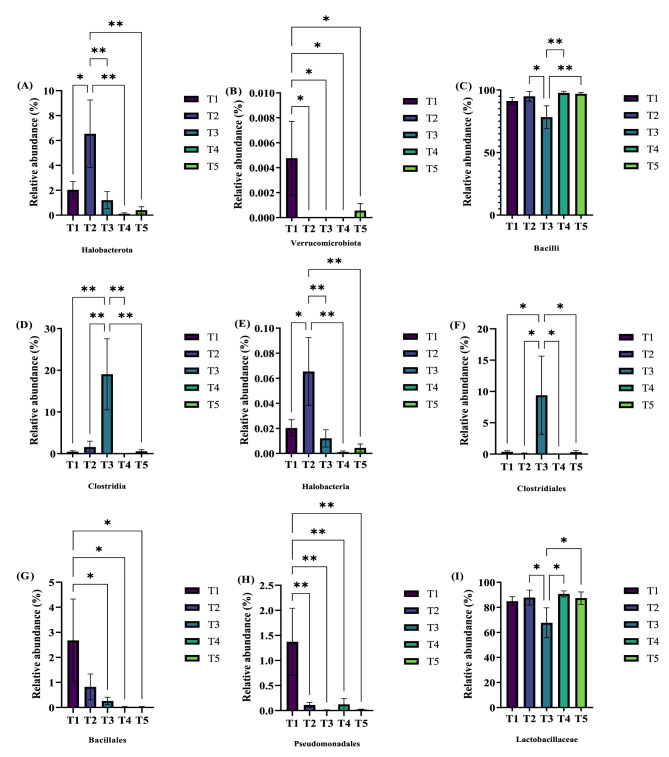
Effects of varying dietary metabolizable energy levels on the relative abundance of fecal flora in exercise-trained pigeons **(A–I)**. Asterisk “*” denotes a significant difference between groups (LDS test, *P* < 0.05), while “**” and higher indicate an extremely significant difference between groups (LDS test, *P* < 0.01). T1: 12.03 MJ/kg dietary metabolizable energy; T2: 12.20 MJ/kg dietary metabolizable energy; T3: 12.32 MJ/kg dietary metabolizable energy; T4: 12.46 MJ/kg dietary metabolizable energy; T5: 12.59 MJ/kg dietary metabolizable energy.

**Figure 6:**
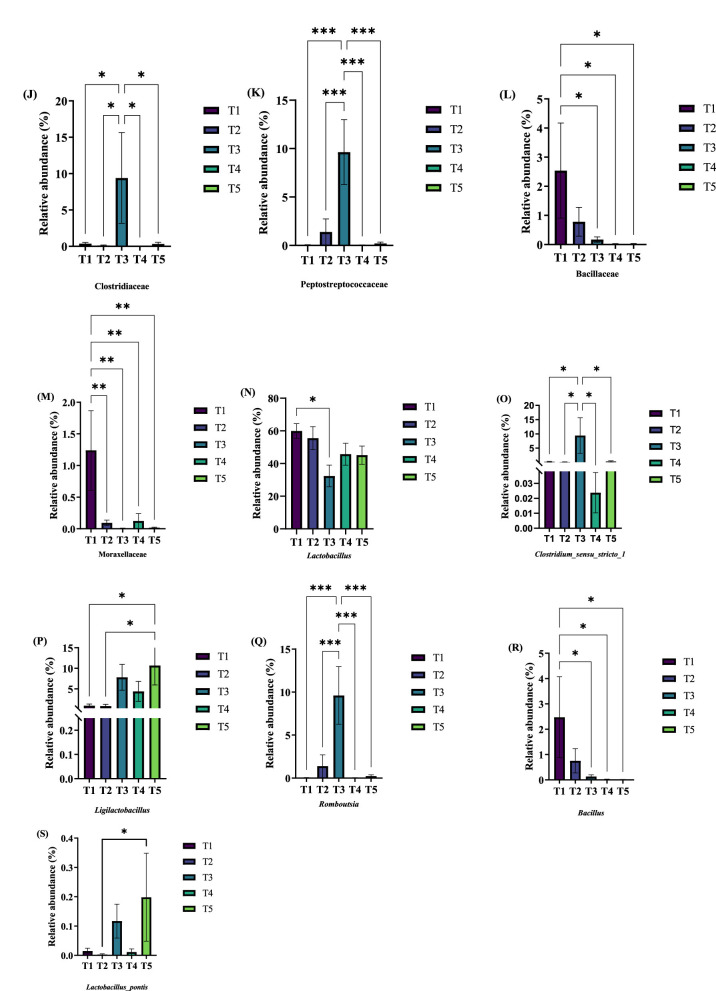
Effects of varying dietary metabolizable energy levels on the relative abundance of fecal flora in exercise-trained pigeons **(J–S)**. **(J)** *Clostridiaceae*, **(K)** *Peptostreptococcaceae*, **(L)** *Bacillaceae*, **(M)** *Moraxellaceae*, **(N)** *Lactobacillus*, **(O)** *Clostridium_sensu_stricto_1*, **(P)** *Ligilactobacillus*, **(Q)** *Romboutsia*, **(R)** *Bacillus*, **(S)** *Lactobacillus_pontis*. Asterisk “*” denotes a significant difference between groups (LDS test, *P* < 0.05), while “**” and higher indicate an extremely significant difference between groups (LDS test, *P* < 0.01). T1: 12.03 MJ/kg dietary metabolizable energy; T2: 12.20 MJ/kg dietary metabolizable energy; T3: 12.32 MJ/kg dietary metabolizable energy; T4: 12.46 MJ/kg dietary metabolizable energy; T5: 12.59 MJ/kg dietary metabolizable energy.

The effects of varying ME levels in the diet on the relative abundance of fecal microbiota in exercise-trained pigeons are shown in Figures 4–6.

At the phylum level, the fecal microbiota compositions were similar across the different ME levels. The top 10 phyla by relative abundance included Firmicutes, Proteobacteria, Actinobacteriota, Bacteroidota, Halobacterota, Cyanobacteria, Deinococcota, Gemmatimonadota, Patescibacteria, and Verrucomicrobiota. Among these, Firmicutes was the dominant phylum across all groups, with abundances of 91.73%, 96.59%, 97.33%, 97.74%, and 97.50%, respectively (Figure 4A). As shown in Figures 5A, B, the relative abundances of Halobacterota (Figure 5A) and Verrucomicrobiota (Figure 5B) were significantly higher in the fecal microbiota of pigeons in group T1 compared to those in groups T2, T3, T4, and T5 (*P* < 0.05).

At the class level, the top 10 classes by relative abundance were Bacilli, Clostridia, Gammaproteobacteria, Actinobacteria, Bacteroidia, Alphaproteobacteria, Coriobacteriia, Negativicutes, Halobacteria, and Cyanobacteriia. Bacilli was the dominant class at this level (Figure 4B). In group T2, at an ME level of 12.32 MJ/kg, the relative abundance of Bacilli (Figure 5C) was significantly lower compared to groups T2, T4, and T5. Furthermore, the relative abundance of Clostridia (Figure 5D) in group T2 was extremely significantly higher than in the other groups (*P* < 0.01). When the ME level was 12.20 MJ/kg, the relative abundance of Halobacteria (Figure 5E) was significantly or extremely significantly higher in group T2 compared to the other groups (*P* < 0.05).

At the order level, the top 10 species by relative abundance were Lactobacillales, Enterobacterales, Clostridiales, Erysipelotrichales, Peptostreptococcales-Tissierellales, Bacillales, Bifidobacteriales, Corynebacteriales, Pseudomonadales, and Sphingobacteriales. Among these, Lactobacillales was the dominant order (Figure 4C). At an ME level of 12.32 MJ/kg, the relative abundance of Clostridiales (Figure 5F) in the fecal microbiota of exercise-trained pigeons was significantly higher than in groups T1, T2, T4, and T5 (*P* < 0.05). The relative abundances of Bacillales (Figure 5G) and Pseudomonadales (Figure 5H) in group T1 were extremely significantly higher than those in groups T3, T4, and T5 (*P* < 0.01).

At the family level, the top 10 species by relative abundance were Lactobacillaceae, Enterobacteriaceae, Clostridiaceae, Enterococcaceae, Erysipelotrichaceae, Peptostreptococcaceae, Bacillaceae, Bifidobacteriaceae, Corynebacteriaceae, and Moraxellaceae. Lactobacillaceae was the dominant family (Figure 4D). The relative abundance of Lactobacillaceae (Figure 5I) in group T4 was significantly higher than in group T3 (*P* < 0.05). The relative abundance of Clostridiaceae (Figure 6J) in group T3 was significantly higher than in groups T1, T2, T4, and T5 (*P* < 0.05). The relative abundance of Peptostreptococcaceae (Figure 6K) in group T3 was extremely significantly higher than in groups T1, T2, T4, and T5 (*P* < 0.01). The relative abundance of Bacillaceae (Figure 6L) in group T1 was significantly higher than in groups T3, T4, and T5 (*P* < 0.05). Finally, the relative abundance of Moraxellaceae (Figure 6M) in group T1 was extremely significantly higher than in groups T2, T3, T4, and T5 (*P* < 0.01).

At the genus level, the top 10 species by relative abundance were *Lactobacillus, Escherichia-Shigella, Clostridium_sensu_stricto_1, Ligilactobacillus, Enterococcus, Turicibacter, Romboutsia, Bacillus, Limosilactobacillus*, and *Aeriscardovia*. *Lactobacillus* dominated the genus level (comprising 59.87%, 55.52%, 32.39%, 45.70%, and 45.11%, respectively; Figure 4E). The relative abundance of *Lactobacillus* in the fecal flora of group T1 (Figure 6N) was significantly higher than that in group T3 (*P* < 0.05). The relative abundances of *Clostridium_sensu_stricto_1* (Figure 6O) and *Romboutsia* (Figure 6Q) in the fecal flora of group T3 were significantly higher than those in groups T1, T2, T4, and T5 (*P* < 0.05). The relative abundance of *Ligilactobacillus* in the fecal flora of group T5 (Figure 6P) was significantly higher than that in groups T1 and T2 (*P* < 0.05). The relative abundance of Bacillus in the fecal flora of group T1 (Figure 6R) was significantly higher than in groups T3, T4, and T5 (*P* < 0.05).

At the species level, the top 10 species by relative abundance were *swine_fecal_bacterium_SD-Pec10, Lactobacillus*_*ingluviei, Aeriscardovia*_*aeriphila, Enterococcus*_*cecorum, Lactobacillus*_*panis, Lactobacillus_agilis, Lactobacillus_pontis, Lactobacillus*_*gigeriorum, Pedobacter_sp_WF1*, and *Galliscardovia*_*ingluviei* (Figure 4F). The relative abundance of *Lactobacillus_panis* in the fecal flora of group T5 (Figure 6S) was significantly higher than in group T2 (*P* < 0.05).

### 3.8 LEfSe analysis of fecal flora in exercise-trained pigeons with different metabolizable energy levels in the diet

**Figure 7:**
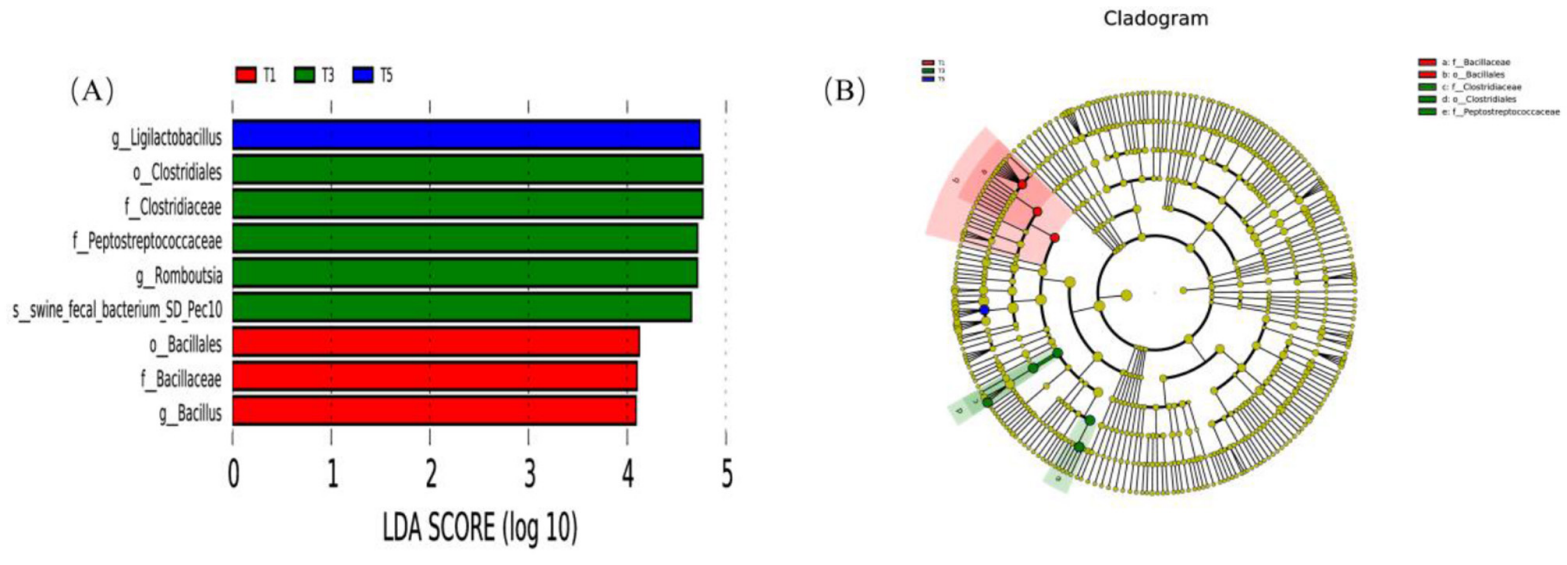
Effects of varying dietary metabolizable energy levels on LEfSe analysis of fecal flora in exercise-trained pigeons. **(A)** Histogram of LDA value distribution. **(B)** Phylogenetic cladogram. T1: 12.03 MJ/kg dietary metabolizable energy; T2: 12.20 MJ/kg dietary metabolizable energy; T3: 12.32 MJ/kg dietary metabolizable energy; T4: 12.46 MJ/kg dietary metabolizable energy; T5: 12.59 MJ/kg dietary metabolizable energy.

Figure 7 presents the LEfSe analysis results of the fecal microbiota in exercise-trained pigeons with varying dietary ME levels. In Group T1, the identified biomarkers include o_Bacillales, f_Bacillaceae, and *g_Bacillus*. For Group T3, the biomarkers are o_Clostridiales, f_Clostridiaceae, f_Peptostreptococcaceae, *g_Romboutsia*, and *s_swine_fecal_bacterium_SD_Pec10*. In Group T5, the sole biomarker is *g_Ligilactobacillus*.

### 3.9 Effects of different metabolizable energy levels in the diet on the functional prediction of fecal flora in exercise-trained pigeons

**Figure 8:**
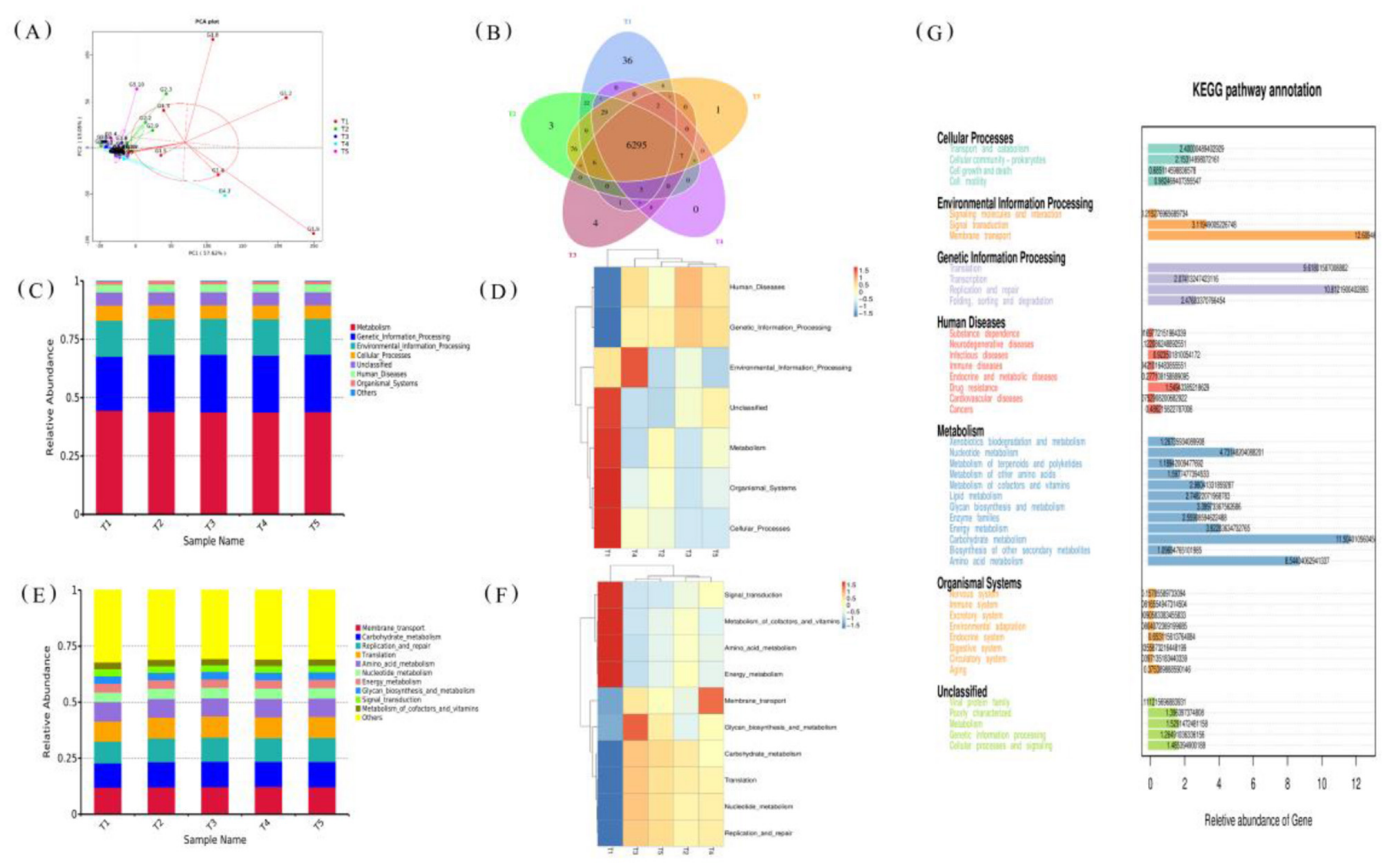
Effects of varying dietary metabolizable energy levels on functional prediction of fecal flora in exercise-trained pigeons. **(A)** β-diversity analysis chart. **(B)** Venn diagram. **(C)** Primary functional prediction chart. **(D)** Primary functional clustering heatmap. **(E)** Secondary functional prediction chart. **(F)** Secondary functional clustering heatmap. **(G)** KEGG (Kyoto Encyclopedia of Genes and Genomes) pathway annotation chart. T1: 12.03 MJ/kg dietary metabolizable energy; T2: 12.20 MJ/kg dietary metabolizable energy; T3: 12.32 MJ/kg dietary metabolizable energy; T4: 12.46 MJ/kg dietary metabolizable energy; T5: 12.59 MJ/kg dietary metabolizable energy.

Figure 8 illustrates the effects of different ME levels in the diet on the functional prediction of the fecal microbiota in exercise-trained pigeons. As depicted, varying energy levels significantly influence the functional profiles of the microbiota. PCA (Figure 8A) reveals that the first principal component accounts for 57.62% of the variance, while the second principal component contributes 13.05%, indicating substantial differences between the groups. Figure 8B shows that Group T1 has 36 unique functions, Group T2 has 3, Group T3 has 4, and Group T5 has 1. Figures 8C, D demonstrate that the microbiota functions in pigeons with different dietary energy levels include Metabolism, Genetic Information Processing, Environmental Information Processing, Cellular Processes, Unclassified, Human Diseases, Organismal Systems, and Others. In Group T1, functions are primarily concentrated in Unclassified, Metabolism, Organismal Systems, and Cellular Processes, whereas Group T2's functions are mainly focused on Environmental Information Processing. Figures 8E,F show that, at the second level, the functions include Membrane Transport, Carbohydrate Metabolism, Replication and Repair, Translation, Amino Acid Metabolism, Nucleotide Metabolism, Energy Metabolism, Glycan Biosynthesis and Metabolism, Signal Transduction, Metabolism of Cofactors and Vitamins, and Others. In Group T1, these functions are concentrated in Signal Transduction, Metabolism of Cofactors and Vitamins, Amino Acid Metabolism, and Energy Metabolism, while Group T3's functions are primarily focused on Glycan Biosynthesis and Metabolism, and Group T4's are centered around Membrane Transport. Figure 8G highlights that the microbiota functions are predominantly enriched in metabolic pathways, with a relatively high proportion (approximately 11.50%) of genes associated with energy metabolism.

### 3.10 Correlation analysis

**Figure 9:**
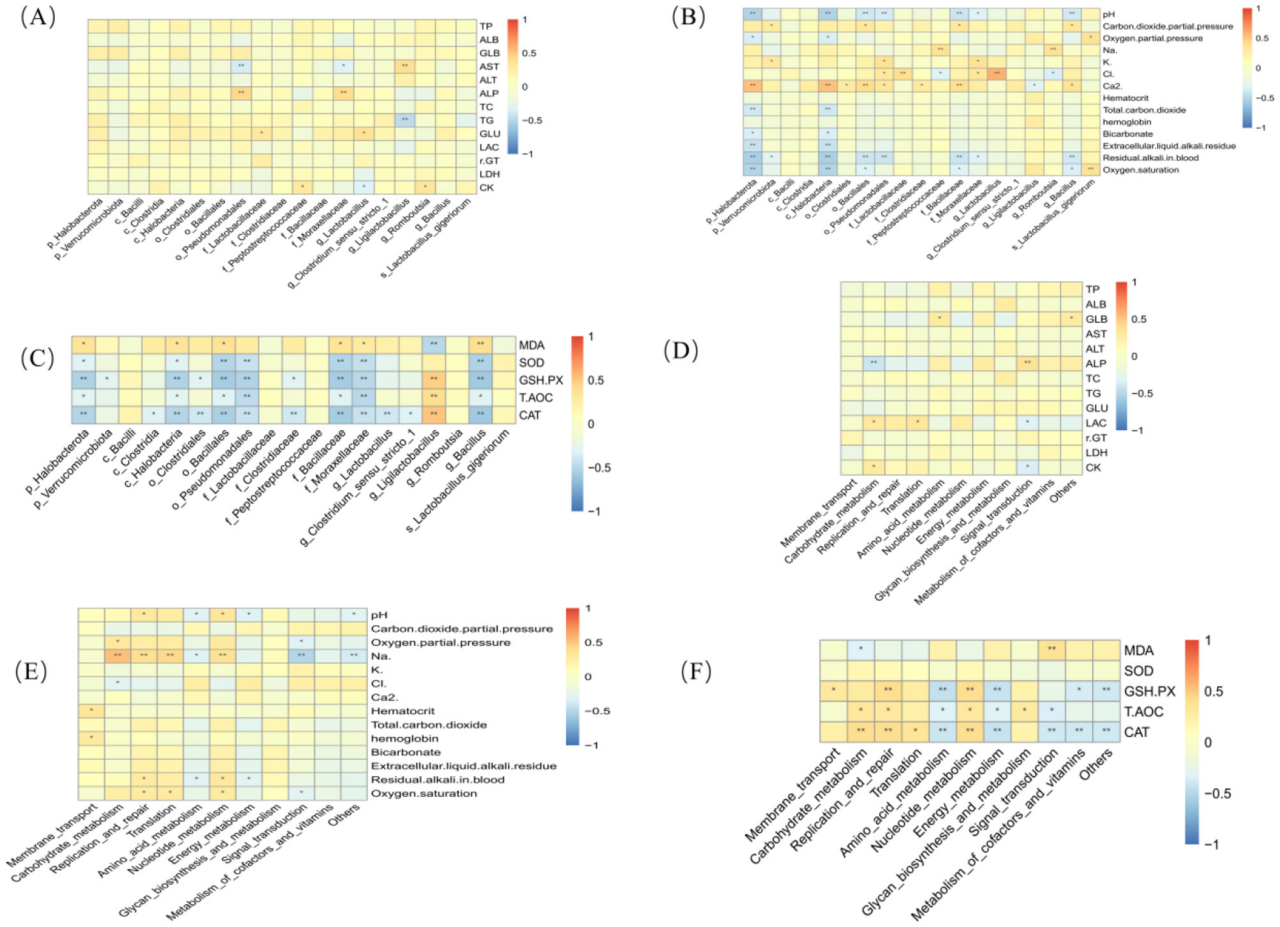
Heatmaps of correlation analysis. Asterisk “*” denotes a significant difference between groups (LDS test, *P* < 0.05), while “**” and higher indicate an extremely significant difference between groups (LDS test, *P* < 0.01). **(A)** Correlation analysis between differential bacteria and blood biochemical indices. **(B)** Correlation analysis between differential bacteria and blood gas indices. **(C)** Correlation analysis between differential bacteria and antioxidant indices. **(D)** Correlation analysis between functional prediction and blood biochemical indices. **(E)** Correlation analysis between functional prediction and blood gas indices. **(F)** Correlation analysis between functional prediction and antioxidant indices.

A Spearman correlation analysis was conducted to investigate the relationships among the fecal microbiota of exercise-trained pigeons, their functions, serum biochemical markers, antioxidant indicators, and blood gas parameters, with the results shown in Figure 9.

Figure 9A reveals that ALP is highly positively correlated with o_Pseudomonadales and f_Moraxellaceae (*P* < 0.01). GLU is strongly positively correlated with f_Clostridiaceae and g_*Lactobacillus* (*P* < 0.01). CK exhibits a significant positive correlation with f_Peptostreptococcaceae and g_*Romboutsia* (*P* < 0.05). Additionally, ALP is highly positively correlated with o_Pseudomonadales and f_Moraxellaceae (*P* < 0.01), and AST is highly positively correlated with g_*Ligilactobacillus* (*P* < 0.01).

Figure 9B indicates that Ca^2+^ is highly positively correlated with p_Halobacterota and c_Bacilli (*P* < 0.01), while Cl is highly positively correlated with f_Lactobacillaceae and g_*Lactobacillus* (*P* < 0.01). Oxygen saturation is strongly positively correlated with s_Lactobacillus_gigeriorum (*P* < 0.01). Furthermore, p_Verrucomicrobiota, o_Bacillales, f_Bacillaceae, and s_Lactobacillus_gigeriorum show significant positive correlations with the partial pressure of CO_2_ (*P* < 0.01).

Figure 9C demonstrates that MDA is significantly positively correlated with p_Halobacterota, c_Halobacteria, o_Bacillales, f_Bacillaceae, f_Moraxellaceae, and g_*Bacillus* (*P* < 0.05).

Figure 9D shows that GLU is strongly positively correlated with Translation (*P* < 0.01), ALP with Signal Transduction (*P* < 0.01), LAC with both Carbohydrate Metabolism and Translation (*P* < 0.01), and CK with Carbohydrate Metabolism (*P* < 0.01).

Figure 9E reveals that Na^+^ is strongly positively correlated with Carbohydrate Metabolism, Replication and Repair, Translation, and Nucleotide Metabolism (*P* < 0.01), while oxygen partial pressure is strongly positively correlated with Carbohydrate Metabolism (*P* < 0.01).

Finally, Figure 9F highlights that CAT is strongly positively correlated with Carbohydrate Metabolism, Replication and Repair, and Nucleotide Metabolism (*P* < 0.01). T-AOC shows a significant positive correlation with Carbohydrate Metabolism, Replication and Repair, and Glycan Biosynthesis and Metabolism (*P* < 0.05). GSH-PX is significantly positively correlated with Membrane Transport, Replication and Repair, and Nucleotide Metabolism. MDA is also significantly positively correlated with Signal Transduction (*P* < 0.05).

## 4 Discussion

### 4.1 Effects of different metabolizable energy levels on the apparent digestion and metabolism of nutrients in athletic training pigeons

The ME and CP levels significantly impact the reproductive and growth performance of pigeons by influencing the digestion and metabolism of dietary nutrients (Peng et al., 2023a,b). Sales and Janssens (2003) demonstrated that adult pigeons are primarily fed whole grain mixtures. The specific feeding characteristics of pigeons limit the applicability of nutrient requirements established for other avian species. It is recommended that adult pigeons be fed a diet containing between 12 and 18% crude protein and approximately 12 MJ/kg of ME, based on offspring production. The apparent ME, corrected for nitrogen retention (AME), for maize (14.76 MJ/kg), barley (12.36 MJ/kg), sorghum (13.87 MJ/kg), and peas (14.01 MJ/kg) did not differ substantially from poultry values. The digestion and metabolism rates of nutrients in poultry vary according to the dietary ME level. Chen (2016) examined the effects of dietary ME and protein levels on growth performance, nutrient utilization, and digestive enzyme activity in Qilin chickens. The study revealed that the utilization rates of crude protein in the high-energy group (ME: 13.80 MJ/kg) and the medium-energy group (ME: 12.75 MJ/kg) were significantly higher than those in the low-energy group (ME: 11.70 MJ/kg; *P* < 0.05). Increasing the ME level in the diet also enhanced crude fat utilization. Yang et al. (2009) explored the effects of varying ME and crude protein levels on nutrient digestibility and growth performance in Hepu geese. The study found that, after 28 days, geese fed diets with 15.10% crude protein and 10.03 MJ/kg ME or 12.50% crude protein and 11.50 MJ/kg ME exhibited high nutrient digestibility. Wang et al. (2024) reported that, at an ME level of 12.6 MJ/kg, increasing the dietary CP had no significant effect on the apparent digestibility of dry matter (DM), organic matter (OM), ether extract (EE), and gross energy (GE) in Tarim pigeons, although the apparent metabolic rate of CP significantly increased or decreased. Appropriate ME levels in the diet are essential for promoting healthy growth in poultry (Chang et al., 2022). Dietary energy influences growth performance, reproductive capacity, and production costs in poultry (Peng et al., 2023a,b). Energy feeds, particularly corn and oil supplements, are major energy sources in poultry diets (Selaledi et al., 2020). Our research primarily focused on corn-soybean meal-based diets, where, under the same CP levels, whether an increase in ME would affect the nutrient digestion and metabolism rates in pigeon diets was investigated. Varying the dietary ME significantly, and in some cases extremely significantly, influenced the apparent digestion and metabolism rates of OM, CP, and EE, consistent with findings from Yang et al. (2009).

### 4.2 Effects of different metabolizable energy levels on serum biochemical indicators of athletic training pigeons

Serum biochemical indicators provide a sensitive reflection of nutrient digestion, absorption, metabolism, and overall health in pigeons (Graugnard et al., 2012; Alves-Bezerra and Cohen, 2017). Protein catabolism leads to elevated blood urea nitrogen (BUN) levels, which are linked to impaired nitrogen retention, CP, and amino acid utilization. Changes in urea nitrogen concentration serve as an indicator of amino acid metabolism and balance in animals (Stanley et al., 2002). In our study, increasing ME levels in the diet resulted in reduced serum TP, which contrasts with findings by Qiao (2013), Chen et al. (2021), and Peng et al. (2023a,b). This suggests that higher energy intake may affect amino acid utilization and conversion, ultimately lowering serum TP levels. The dietary energy-to-egg ratio influences metabolic processes and overall health. While dietary protein remained constant in our study, the increase in energy levels could explain the observed reduction in serum TP. Furthermore, previous studies by Guan et al. (2017) and Ozek and Bahtiyarca (2004) have shown that higher dietary protein levels enhance protein synthesis in organisms. Despite a 17.1% protein inclusion in the diet, the low serum TP content may reflect an increase in protein synthesis, leading to a reduction in free TP. Conversely, excessive dietary protein intake has been associated with increased protein catabolism, resulting in higher UA synthesis or excretion (Darsi et al., 2012; Liu et al., 2016). However, as serum UA levels were not measured in our study, the potential role of UA metabolism in the observed reduction of serum protein remains unexplained. Lipids encompass a range of lipid substances in the blood, primarily including cholesterol and triglycerides, with their levels serving as direct indicators of lipid absorption and metabolism (Guan et al., 2014). Triglycerides, as the primary form of fat storage, play a key role as an energy source and are involved in cholesterol synthesis. High-density lipoprotein (HDL) and low-density lipoprotein (LDL) are the primary carriers of cholesterol. Liu et al. (2016) reported that energy levels did not significantly affect serum cholesterol, triglyceride, LDL, or HDL levels in Sichuan white geese during early perinatation. In contrast, Peng et al. (2023a,b) observed that, under the same dietary protein levels, plasma TG levels in breeding pigeons decreased with increasing dietary metabolic energy, while those in squabs increased. In our study, an increase in ME, with constant dietary protein, led to elevated serum TG content in pigeons, aligning with findings by Qiao (2013) and Chen et al. (2021). This suggests that protein levels influence amino acid utilization and conversion in squabs, thereby elevating plasma TG levels. Furthermore, since the increased TG content remained within normal ranges, higher dietary energy levels appeared to have a beneficial impact on fat metabolism in pigeons.

ALP plays a role in fat metabolism, with some studies indicating a correlation between ALP activity and growth rate, as well as daily weight gain. Glutamic pyruvic transaminase (GPT) and glutamic oxalacetic transaminase (GOT) primarily reflect liver function. In our study, ME levels in the diet had no significant effect on serum ALT and ALP activities, consistent with findings by Fang (2022), but differing from results by Guan et al. (2014). These discrepancies may be attributed to differences in energy levels, poultry species, and feeding stages. ALP plays a role in fat metabolism. Some studies have shown that there is a correlation between alkaline phosphatase activity and growth rate as well as daily weight gain. This study found that ME levels significantly altered serum GLU and TG. Increasing the intake of metabolic energy can promote hepatic gluconeogenesis and raise serum glutamic acid levels (Lu et al., 2022). Alanine aminotransferase (ALT) and aspartate aminotransferase (ALP) mainly reflect liver function. In our study, as the metabolic energy level in the diet increased, the activities of serum ALT and ALP also rose accordingly. Studies have shown that after LDHA-BB genotype racing pigeons were fed a high metabolic energy diet, their muscle lactic acid concentration was 3.2 times that of AA genotype racing pigeons, and the AST activity was positively correlated. High metabolic energy diet causes muscle lactic acid accumulation in pigeons with LDHA-AB/BB genotype. Elevated serum AST/ALT levels reflect the risk of liver injury (Kolvenbag et al., 2022). It is consistent with the research results of Fang (2022), but different from those of Guan et al. (2014). These differences may be related to the influence of energy levels, poultry species, rearing stage and training load.

### 4.3 Effects of different metabolizable energy levels on the serum antioxidant capacity of athletic training pigeons

Under normal metabolic conditions, low levels of reactive oxygen species (ROS) play a role in various biochemical pathways and physiological processes (Cheng et al., 2023a,b). However, excessive ROS production can lead to oxidative stress, damaging biological macromolecules such as lipids, proteins, and nucleic acids, and resulting in the accumulation of harmful byproducts like MDA, a marker of lipid peroxidation. This further exacerbates oxidative damage to cells and tissues (Mayor Oxilia, 2010). The body also contains antioxidant systems, including SOD, GSH-Px, and CAT, which are crucial for maintaining redox homeostasis (Cheng et al., 2023a,b). GSH-Px catalyzes the disproportionation of superoxide anion radicals into hydrogen peroxide, which is then broken down into water and oxygen through the combined action of GSH-Px and CAT (El-Senousey et al., 2018). Previous studies have shown that high-energy diets can induce oxidative stress (Wang, 2010; Pedernera et al., 2010; Tan and Norhaizan, 2019). Niu (2024) demonstrated that MDA content in the jejunum of squabs increased linearly with dietary energy level, indicating a state of oxidative stress and enhanced lipid peroxidation. The increased energy levels also led to a linear rise in the activity of GSH-Px in the jejunum and T-SOD in the ileum, suggesting a self-regulatory mechanism in livestock and poultry. The upregulation of antioxidant enzyme activity may help regulate oxidative stress and mitigate the damage caused by excessive ROS. In our study, increasing ME levels in the diet resulted in elevated levels of SOD, CAT, GSH-Px, and T-AOC in pigeon serum, supporting the findings of Peng et al. (2023a,b). Peng et al.'s (2023a,b) study systematically evaluated the impact of dietary nutrient levels on antioxidant substances (e.g., SOD, CAT) and oxidative damage markers (e.g., MDA) in plasma. It was found that dietary energy and protein levels had minimal effect on oxidative stress indicators in female pigeons, but with increasing dietary CP levels, H_2_O_2_ content in male pigeon plasma rose significantly, and SOD activity decreased, indicating oxidative stress due to excessive protein intake. This study also showed that squabs' antioxidant capacity was significantly improved at higher protein levels, likely due to their higher protein requirements during early development. Most young animals, in their rapid growth phase, have considerably higher protein demands for cellular maintenance and differentiation (Maliwan et al., 2019; Tokach et al., 2019). The lower metabolic energy levels in our study, compared to those in Peng et al.'s, suggest that a lower energy diet, while still meeting the pigeons' energy requirements, offers a better protective effect against oxidative stress.

### 4.4 Effects of different metabolizable energy levels on blood gas of athletic training pigeons

The body requires oxygen for internal oxidation, primarily for energy metabolism. In anaerobic or hypoxic conditions, energy production is incomplete. During oxygen utilization, CO_2_ is produced and excreted from the body. This O_2_ consumption and CO_2_ production process is reliant on the body's gas exchange system, with blood playing a pivotal role in facilitating this exchange. Blood gas analysis refers to the measurement of O_2_ and CO_2_ involved in blood gas exchange, as well as parameters related to acid-base balance. Through analysis, the body's ventilation and gas exchange functions, along with potential acid-base imbalances, can be assessed (Luo and Feng, 1997). Blood gas analysis helps evaluate the internal state of livestock, particularly regarding gas exchange, acid-base balance, and oxygenation. Furthermore, metabolic processes such as the maintenance of osmotic pressure, regulation of acid-base balance, and cardiac and muscle function are tightly linked to electrolyte levels. A stable internal environment is essential for normal cellular metabolism and healthy growth in livestock (Jensen and Bech, 1992). Healthy animals maintain a balanced acid-base status, but certain adverse factors can disrupt this balance, leading to physiological dysfunctions that impair production performance. Blood gas indicators provide a rapid and precise reflection of changes in internal acid-base conditions and are critical for monitoring the body's acid-base balance. Research on blood gas typically focuses on changes before and after exercise, the effects of high altitude, heat stress, disease impact, and differences between arterial and venous blood gases. In poultry, studies often address heat stress in chickens, while research on pigeons tends to focus on transportation stress, shallow hypothermia, and shivering due to external and central cooling. In this study, increasing the levels of ME in the diet resulted in higher SvO_2_, Na^+^, Ca^2+^, anion gap, hemoglobin, and hematocrit levels in pigeon serum, while reducing PvCO_2_.

### 4.5 Effects of different metabolizable energy levels on the diversity of fecal flora in athletic training pigeons

The avian gut microbiota comprises trillions of microorganisms, primarily bacteria, within a dynamic ecosystem that shares a fundamentally symbiotic functional association with the host. It acts as a strategic manager of host physiology involved in regulating avian health (Oakley et al., 2014; Stanley et al., 2014). The microbiome can potentially influence poultry physiology by participating in digestion, nutrient absorption, shaping mucosal immune responses, energy homeostasis, and the synthesis or modulation of several potentially bioactive metabolites (Kogut, 2022). The species composition and metabolic functions of the gut microbial community are readily altered by diet, antibiotic intake, and other host-environment-dependent events. Furthermore, different dietary patterns (e.g., high-fiber, high-energy diets) can significantly alter gut microbiota structure and function (Sanz et al., 2025).

The types and abundance of gut microbiota are critically related to intestinal (or overall body) health. Exercise training and dietary nutritional levels can intervene in gut microbiota composition and abundance, thereby affecting material metabolism. Han et al. (2020) studied rowing athletes and found that the elite group (AE/YE), characterized by a *Prevotella*-dominant enterotype (Enterotype 1), enriched branched-chain amino acid metabolic pathways, significantly enhancing muscle repair efficiency; the non-elite group (YN) was dominated by carbohydrate-degrading bacteria with lower energy conversion efficiency. Martin et al. (2025) further revealed that the gut microbiota of high-performance individuals (dominated by *Prevotella*) exhibited increased expression of SCFAs synthesis genes, particularly enhanced propionic/valeric acid production pathways, directly improving insulin sensitivity and muscle glycogen storage in mice. Diet interacts with the gut microbiota, directly promoting or inhibiting its growth and altering the gut lumen microenvironment, thereby indirectly changing host metabolism and the immune system. Bacteria associated with SCFAs production influence the body's health through the “gut microbiota-gut-brain axis” regulatory model (Dohnalová et al., 2022).

Our study found that at a dietary metabolizable energy (ME) level of 12.32 MJ/kg, the abundance of *Clostridiales, Peptostreptococcaceae*, etc., in racing pigeon feces significantly or highly significantly increased, indicating that dietary energy level can alter gut microbiota abundance. Simultaneously, these changes in microbial abundance are related to the optimal dietary metabolizable energy requirement for training pigeons. Neither excessively high nor excessively low dietary ME levels produced this increase in abundance for the aforementioned bacteria. Research has found that these bacteria primarily metabolize in the animal gastrointestinal tract to produce SCFAs; for instance, metabolites of carbohydrate metabolism like acetate and butyrate can alter gut permeability, thereby improving intestinal and systemic anti-inflammatory, antioxidant, and immune functions. Within the body, they can inhibit LPS-mediated inflammatory responses, subsequently enhancing the activity of the body's antioxidant enzymes (e.g., increased SOD) (Vijay et al., 2021).

Our research found that when dietary ME was above 12.32 MJ/kg, serum levels of AST, ALT, and ALP increased, playing a role in maintaining normal liver and kidney function. Furthermore, the body's serum antioxidant enzymes GSH-PX, SOD, CAT, and T-AOC significantly or highly significantly increased with rising dietary ME levels, while MDA significantly or highly significantly decreased. Butyrate or butyric acid is the primary energy source for colonic epithelial cells and has been shown to be crucial for colonic cell homeostasis and the development of intestinal villus morphology. It can improve the growth performance and carcass quality characteristics of poultry and increase intestinal barrier integrity (Yeoman et al., 2012). This indicates that dietary metabolizable energy level can regulate the composition and abundance of gut microbiota, increase the production of metabolites beneficial to body health, and thereby improve the body's health status.

Endogenous microorganisms in the gastrointestinal (GI) tract significantly influence animal health, playing key roles in nutritional, metabolic, physiological, and immunological processes (Lan et al., 2005; Gerritsen et al., 2011; Waite and Taylor, 2014). The population density and composition of intestinal microbes continuously change in response to factors such as diet (Lu et al., 2003), age (Amit-Romach et al., 2004), disease, and the specific location within the GI tract (Ranjitkar et al., 2016). These microorganisms are integral to host-related metabolic activities and can alter gut morphology, which in turn affects nutrient digestion, absorption, and feed conversion, ultimately regulating animal growth and metabolism (Pan and Yu, 2014). Studies have shown that dietary nutritional levels impact the composition of the intestinal microbiota in monogastric animals, thereby influencing intestinal physiological functions (Cummings and Macfarlane, 1997). The quantity and structure of the intestinal microbiota are pivotal in the animal's nutritional metabolism and immune function (Lee and Hase, 2014). Under normal conditions, changes in the intestinal microbiota are primarily driven by factors such as health status, feed type, and environmental conditions. Research indicates that diet has the most pronounced effect on the quantity and composition of the intestinal microbiota (Trotter, 1997; Zhang et al., 2010a,b). While utilizing dietary energy, the intestinal microbiota ferments and decomposes substances like crude fiber and crude protein, obtaining nutrients and providing energy for the animal (Ramayo-Caldas et al., 2016). Thus, diet directly influences the composition and quantity of the intestinal microbiota. In a study on Dezhou Sanfen female donkeys, Yue (2022) found that a dietary digestible energy (DE) level of 13.10 MJ/kg significantly reduced the Sob, ACE, Chao, and Shannon indices of the rectal microbiota, while the Simpson index increased. This suggests that an excessively high dietary energy level decreases the abundance and diversity of rectal bacteria in lactating donkeys compared to those on diets with lower energy levels (12.40 MJ/kg). Several studies on herbivores have yielded similar findings. Yao et al. (2022) and Zhang et al. (2021) reported that high-energy diets significantly reduced the Chao and ACE indices of yak rumen microbiota. Park et al. (2020) observed that a low-energy diet increased both the abundance and species diversity of rumen flora in dairy cows. Wu (2012) found that appropriately elevated dietary energy levels led to a more complex dominant microbiota in the intestines of Rex rabbits; however, excessive dietary energy resulted in a marked reduction in microbial diversity. Yang (2024) investigated Tunchang black pigs and found no significant changes in the α-diversity indices of the ileum as dietary net energy levels decreased (10.65, 10.15, 9.65, and 9.15 MJ/kg). However, in the colonic contents, the Chao1, Shannon, Observed species, and Pielou's evenness indices were significantly higher in the 9.65 MJ/kg group compared to the 10.15 MJ/kg group. This effect may be attributed to the lower fiber content of high-energy diets. Li et al. (2015) suggested that low-energy, high-fiber diets enhance rumen flora diversity. *Firmicutes, Bacteroidetes*, and *Proteobacteria* have been identified as the predominant phyla in the gastrointestinal tracts of poultry (Best et al., 2016; Wang et al., 2018; Stanley et al., 2013; Deusch et al., 2015). In chickens, *Firmicutes (*~78%) and *Proteobacteria (*~16%) predominated in the crop, while *Firmicutes (*~50%), *Bacteroidetes (*~29%), and *Actinobacteria (*~10%) were dominant in the caecum (Saxena et al., 2016). In avian species and pigeons, Firmicutes, Proteobacteria, and Actinobacteria were the primary phyla in the ileum, constituting 98.02% of the microbiota (Sun et al., 2023; Khan et al., 2020). Our findings indicate that increasing ME levels in the diet reduced both the Chao1 and observed features indices of fecal samples, corroborating results observed in the fecal microbiota. Firmicutes accounted for over 90%, which contrasts with Maurine W. Dietz's results, where Firmicutes represented 43.1% ± 17.7 of pigeon fecal samples (Yao et al., 2022; Zhang, 2021). Previous studies on herbivorous livestock have shown that reducing dietary DE from 11.90 to 10.43 MJ/kg increased the relative abundance of Firmicutes, the dominant phylum in Dezhou donkey rectal flora (Zhang et al., 2021). In a study of Tunchang black pigs' ileum contents, Firmicutes emerged as the most abundant phylum, with dietary energy levels of 10.15 and 9.65 MJ/kg significantly elevating its relative abundance (Yang, 2024). During migration, fasting-induced stress in migratory birds results in an increased proportion of Firmicutes in the gut, potentially enhancing energy intake efficiency (Turjeman et al., 2020). Research suggests that Firmicutes genes encode numerous enzymes involved in energy metabolism, enabling the digestion of various substances (Kaakoush, 2015). An increase in the Firmicutes/Bacteroidetes (F/B) ratio can improve fat metabolism and enhance intestinal nutrient absorption and energy conversion (Bäckhed et al., 2005). Thus, this shift in gut microbiota composition benefits the host's nutrient digestion and absorption, enhancing energy metabolism efficiency (Kaakoush, 2015). Consequently, the observed increase in Firmicutes abundance in response to higher dietary ME levels is linked to improved energy metabolism efficiency in pigeons.

The increase in dietary ME was associated with a significant or highly significant decrease in the relative abundances of *Halobacteria, Bacillaceae, Moraxellaceae, Bacillales, Pseudomonadales, Lactobacillus*, and *Bacillus*. At an ME level of 12.20 MJ/kg, there was a notable increase in the relative abundances of Clostridia, Peptostreptococcaceae, Clostridiales, Peptostreptococcales-Tissierellales, Clostridium_sensu_stricto_1, Romboutsia, and swine_fecal_bacterium_SD-Pec10. The observed reduction in *Lactobacillus* abundance aligns with the findings of Nikravesh-Masouleh et al. (2018), who reported that higher dietary energy levels lead to decreased *Lactobacillus* populations. In contrast, a decrease in ME from 10.88 MJ/kg to 10.04 MJ/kg resulted in a significant increase in Lactobacillus relative abundance in ostrich intestinal flora (Nikravesh-Masouleh et al., 2018). Wang et al. (2023) reported that a high-fat diet increased gut microbiota diversity, enriching Desulfovibrionaceae, Rikenellaceae RC9, and Spirochaetes, while reducing Lactobacillus, Bifidobacterium, Akkermansia, Faecalibaculum, and Blautia. At the genus level, differential microbiota among energy groups primarily included Colidextribacter, norank f_Prevotellaceae, and Butyricimonas. Colidextribacter, which produces inosine, provides a physiological energy source and possesses anti-inflammatory and immunomodulatory properties, such as inhibiting pro-inflammatory factor and chemokine production while promoting anti-inflammatory cytokine production (Guo et al., 2021). Proteobacteria, the largest bacterial phylum and primarily pathogenic, has a positive correlation with the incidence of diseases such as obesity and diabetes; its increased levels are often associated with metabolic disorders (Rizzatti et al., 2017; Moon et al., 2018). Li et al. (2024) observed a gradual decrease in Proteobacteria abundance with decreasing dietary ME levels. In contrast, our study found a decrease in Proteobacteria abundance in pigeon feces with increasing ME levels, differing from the results of Li et al. (2024). This suggests that an optimal dietary ME level can enhance the relative abundance of beneficial genera and reduce harmful genera, promoting the healthy development of the intestinal microbiota in pigs. Previous studies have also shown that dietary energy levels influence intestinal health by altering microbiota composition, with both excessive and insufficient energy levels leading to changes in intestinal microbiota composition (Zhang et al., 2023).

LDA effect size (LEfSe) (Segata et al., 2011) is a robust analytical tool for identifying and interpreting high-dimensional biological markers, enabling comparisons across multiple groups. It emphasizes both statistical significance and biological relevance, facilitating the identification of biomarkers that exhibit significant differences between groups. The analysis begins with the Kruskal–Wallis rank sum test to identify all characteristic species by assessing differences in species abundance across groups. Species with significant differences are then selected. Subsequently, the Wilcoxon rank sum test is applied to determine whether the significant species identified previously converge at the same taxonomic level. Finally, linear discriminant analysis (LDA) is performed to pinpoint the final differential species (i.e., biomarkers). In our study, nine differential bacteria were identified using a threshold of 4. At a dietary ME level of 12.03 MJ/kg, Bacillalesf, Bacillaceae, and Bacillus were identified, while at an ME level of 12.32 MJ/kg, Clostridiales, Clostridiaceae, Peptostreptococcaceae, Romboutsia, and Swine_fecal_bacterium_SD_Pec10 were found. At an ME level of 12.59 MJ/kg, *Ligilactobacillus* was identified. *Ligilactobacillus*, a lactic acid bacterium, is a member of the probiotic group and plays a significant role in the gastrointestinal tract of animals (Boll et al., 2024). *Ligilactobacillus salivarius*, a strain of *Ligilactobacillus*, is an important constituent of the gastrointestinal tract (GIT). Certain strains of *L. salivarius* are known for their beneficial effects on the host, particularly through the production of antimicrobial peptides that help maintain a healthy gut microbiota. The ability of *Ligilactobacillus* to inhibit the proliferation of harmful bacteria is consistent with the results observed in our study. As dietary ME levels increased, Proteobacteria abundance decreased. This may be due to the high ME levels promoting *Ligilactobacillus* growth, which in turn inhibits the expansion of Proteobacteria, thereby supporting intestinal health. Intestinal microbiota plays a critical role in the body's fat metabolism, with colonic microorganisms converting incompletely digested carbohydrates into short-chain fatty acids (SCFAs). The probiotic effects of butyric acid, a SCFA, are particularly prominent. Butyrate-producing bacteria, such as Clostridiaceae, Peptostreptococcaceae, and Pasteurellaceae (Wang et al., 2023), were identified in our study at an ME level of 12.32 MJ/kg, with significant differences in Clostridiales, Clostridiaceae, Peptostreptococcaceae, and Romboutsia. This indicates that an optimal dietary ME level can promote the degradation of carbohydrates in pigeon diets, leading to the production of abundant SCFAs and providing abundant energy for pigeons.

Tax4Fun functional prediction utilizes the nearest neighbor method based on a minimum 16S rRNA sequence similarity. The process involves extracting prokaryotic genome 16S rRNA gene sequences from the KEGG database and aligning them to the SILVA SSU Ref NR database using the BLASTN algorithm (BLAST bitscore >1,500) to establish a correlation matrix. Functional annotations from the KEGG database, as identified by the UProC and PAUDA methods, are then mapped to the SILVA database, enabling functional annotation of the SILVA sequences. Sequencing samples are clustered into OTUs with SILVA database sequences as references, from which functional annotation data is derived. In our study, the predicted bacterial functions primarily span seven categories: Metabolism, Genetic Information Processing, Environmental Information Processing, Cellular Processes, Unclassified, Human Diseases, and Organismal Systems. Metabolism is the predominant function in pigeon feces, encompassing 12 metabolic pathways, including Xenobiotics biodegradation and metabolism, Nucleotide metabolism, Terpenoid and polyketide metabolism, Other amino acid metabolism, Cofactor and vitamin metabolism, Lipid metabolism, Glycan biosynthesis and metabolism, Enzyme families, Energy metabolism, Carbohydrate metabolism, Secondary metabolite biosynthesis, and Amino acid metabolism. Notably, Carbohydrate metabolism (11.50%) and Amino acid metabolism (8.54%) pathways exhibit the highest gene enrichment. Previous research has shown that host-ingested carbohydrates are fermented by gut microbiota into metabolites, primarily composed of SCFAs like acetic acid, propionic acid, and butyric acid, which regulate energy intake (Krautkramer et al., 2020). In our study, increasing the dietary ME level elevated carbohydrate intake, which in turn influenced the metabolism of Clostridiales, Clostridiaceae, Peptostreptococcaceae, and Romboutsia. This enhanced carbohydrate metabolism and regulated the energy metabolism of pigeons. Furthermore, the enrichment of functional pathways such as Nucleotide metabolism and Amino acid metabolism highlights the involvement of gut microbiota in the body's energy metabolism processes.

## 5 Conclusions

Dietary ME level significantly influenced the nutrient digestion and metabolism, serum biochemical indices, as well as microbial diversity and composition in sport pigeons. As pigeons' dietary energy intake levels increased, this increase strongly affected nutrient requirements, health, and gastrointestinal tract balance.

In nutrient digestion and metabolism:

At a dietary ME of 12.59 MJ/kg, digestibility of OM and EE significantly increased; at a dietary ME level of 12.32 MJ/kg, CP digestibility significantly improved.

In serum biochemical indices:

TP and GLB levels were significantly higher at a dietary ME of 12.03 MJ/kg than at other dietary ME levels; AST, ALT, and ALP levels increased when dietary ME exceeded 12.32 MJ/kg.

In microbial diversity:

At a dietary ME of 12.03 MJ/kg, the chao1 index and observed_features index were optimal; at a dietary ME of 12.46 MJ/kg, the pielou_e index was significantly higher than at lower ME levels (12.03 and 12.20 MJ/kg);Furthermore, increased dietary ME levels enhanced carbohydrate intake, which in turn influenced the metabolism of *Clostridiales, Clostridiaceae, Peptostreptococcaceae*, and *Romboutsia*, thereby regulating the energy metabolism of pigeons.

In serum antioxidant capacity:

The body's serum antioxidant enzymes GSH-PX, SOD, CAT, and T-AOC significantly or highly significantly increased with rising dietary ME levels, while MDA significantly or highly significantly decreased.

Within the energy level range of 12.32–12.46 MJ/kg, optimal production performance, apparent nutrient digestibility, pigeon health, antioxidant capacity, gut microbiota diversity, and microbial abundance were achieved.

## Data Availability

The data provided in the study is stored in the NCBI repository (https://www.ncbi.nlm.nih.gov), accession number PRJNA1309695.
